# Pharmacokinetics study of bio-adhesive tablet of *Panax notoginseng *saponins

**DOI:** 10.1186/1755-7682-4-18

**Published:** 2011-06-09

**Authors:** Hanzhou Feng, Wei Chen, Chunyan Zhu

**Affiliations:** 1 Department of Natural Medicine Chemistry Research Center, Institute of Medicinal Plant Development, Chinese Academy of Medical Sciences and Peking Union Medical College, No.151 Malianwa North Road, Beijing, 100193, P.R. China

## Abstract

Panax notoginseng saponin (PNS) is the main active gradient of Chinese traditional medicine Panax notoginseng. Although its prominent therapeutic efficacy has been demonstrated by various researchers, the broader application is restricted by the low bioavailability of PNS. This article aims to discuss PNS's plasma pharmacokinetics after oral administration of bio-adhesive tablet of PNS to beagle dogs and improve its bioavailability in comparison with normal tablet. The bio-adhesive tablet was prepared according to our previous patent, using chitosan as main excipient. A simple and sensitive LC-MS/MS combined with solid-phase extraction (SPE) method for the analysis of PNS in dog's plasma was developed in our previous study, and was validated to apply in the pharmacokinetics study in this work. Three ingredients: Notoginsenoside R1 (R1), Ginsenoside Rg1 (Rg1) and Ginsenoside Rb1 (Rb1) (Figure 1), were chosen as indicators of PNS to analyze it in vivo. Statistically significant increase (P < 0.05) in pharmacokinetic parameters of PNS including AUC and Tmax for R1, Rg1 and Rb1, Cmax for R1 and Rb1, MRT for Rg1 were obtained after oral administration of bio-adhesive tablet of PNS comparing with its normal tablet. The formulation modification of using chitosan to prepare bio-adhesive tablet for oral administration is effective in improving the bioavailability of PNS, thereby enhancing its potential therapeutic effect and broadening its clinical application.

## 1. Introduction

*Panax notoginseng *(Burk.) F.H. Chen is an important traditional Chinese medicine mainly produced in Yunnan, Guangxi and Szechwan in China.*Panax notoginseng *saponin (PNS), the extract of *Panax notoginseng*, has been generally studied. PNS possesses many pharmacological active effects, including resistance against cerebral infarction and ischemia by inhibiting apoptosis and expression of caspase-1 and caspase-3 [1,2]; antioxidant property by its strong ferrous ion chelating activity scavenging activities against hydrogen peroxide, hydroxyl radicals, superoxide anion and DPPH free radicals [3]; therapeutic effect on atherosclerosis by lowering serum lipid level and regulating expression of vascular cell differentiation antigen 40 and matrix metalloproteinase 9 [4]. In recent years, increasing attention has been paid to other activities of PNS: neuroprotective activity based on its potent protective effect against blood-brain barrier damage) [5]; anti-hyperglycemic and anti-obese effects by lowering fasting blood glucose levels and improving glucose tolerance [6]; anti-platelet and anti-coagulant effects [7]; anti-inflammatory in vitro properties via blocking NF-κB signaling pathway in macrophages [8]; anti-depressive activity that may be mediated by modulation of brain monoamine neurotransmitters and intracellular Ca2+ concentration (Xiang et al.: The antidepressant effects and mechanism of action of total saponins from the caudexes and leaves of Panax notoginseng in animal models of depression, submitted).

Currently, there are some commercial formulations of PNS in China, including XUESAITONG tablet, injection and lyophilized powder injection. However, the oral bioavailability of PNS is not satisfying, and it is documentarily reported that some adverse reactions such as epistasis, allergy and even anaphylactic shock might be caused by using PNS injection [9]. In order to avoid such serious adverse effects as well as to improve the oral bioavailability of PNS, developing more appropriate formulation is urgently needed. In our lab's previous studies, we applied a widely used bio-adhesive excipient, carbopol, to make bio-adhesive tablet [10]. However, chitosan is a more bio-compatible excipient, it has been reported that the median lethal dose (LD50) for mice after oral administration of chitosan is 16 g/kg. Thus the toxicity of chitosan is considerably lower than carbopol, the LD50 of which is 2.5 g/kg [11]. Based on the consideration of choosing an excipient with lower toxicity, in this study we used chitosan to prepare bio-adhesive tablet.

**Figure 1:**
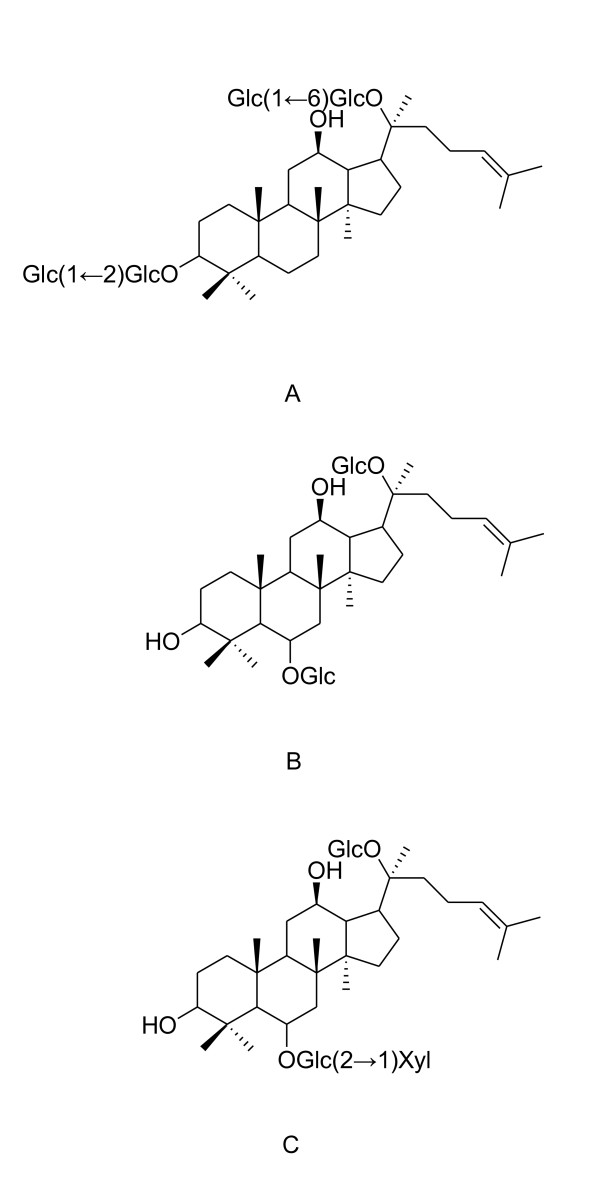
**Chemical structures of Ginsenoside Rb1 (A), Ginsenoside Rg1 (B) and Notoginsenoside R1 (C)**.

Researchers have done some pharmacokinetics studies of PNS [12,13], most of these studies chose Ginsenoside Rg1 or Ginsenoside Rb1 as indicator of PNS for analyzing. Although reported detection method simultaneously analyzed Notoginsenoside R1, Ginsenoside Rg1, Rd, Re and Rb1 in rat plasma by HPLC/ESI/MS [14], their sample preparation method using liquid-liquid extraction is different from our solid-phase extraction (SPE) method. In our study, we adopted a liquid chromatography-tandem mass spectrometry (LC/MS/MS) combined with SPE method established in our previous study to simultaneously detect Notoginsenoside R1, Ginsenoside Rg1 and Rb1 to reflect the veritable pharmacokingetics properties of PNS in plasma sample of beagle dogs [15]. The chemical structures of the three components are shown in Figure 1. It is expected that this research would be effective for improving bioavailability of PNS and their clinical therapeutic efficacy, and can serve as a significant foundation for further pharmacological studies of PNS.

## 2. Experimental

### 2.1. Instruments

Blood samples were pretreated with extract-clean C18 (solid-phase extraction) cartridge columns (1 mL, packed with 100 mg of 40 μm octadecyl silica) (Alltech Company, Deerfield, USA). The assay was performed on an Agilent 1100 series HPLC system (Agilent Technologies, Waldbronn, Germany) consisting of a quaternary pump, a triple quadrupole mass spectrometry detector, an autosampler, a vacuum Degasser and a column oven. The mass spectrometer (ABI, USA) equipped with a turboIonspray source was used for the analysis. The mobile phase with the flow rate of 0.2 ml/min consisting of 10 mM ammonium acetate solution, 0.1% n-butyl-amine, 10% methanol in water (A) and methanol (B). The system was run by a gradient program of 50% B to 90% B in five minutes and 90% B to 50% B in three minutes. Single-punch tablet press (TDP, Tianxiang pharmaceutical machinery factory, Shanghai)

### 2.2. Materials

PNS extract was purchased from the Institute of Medicinal Plant Development, Yunnan Branch (Yunnan Province, China). PNS extract contained 37% of Rg1, 36% of Rb1, and 10% of R1, respectively, according to HPLC analysis. Standards of R1, Rg1 and Rb1 were purchased from the National Institute of the Control of Pharmaceutical and Biological Products (Beijing, China). In addition, Glipizide (internal standard) was generously provided by Professor Jifen Guo at the Academy of Military Medical Sciences (Beijing, China). Chitosan was purchased from Jinqiao Biochemistry Company (Zhejiang, China). Acetonitrile and methanol (HPLC grade) were purchased from Fisher Scientific (New Jersey, USA). The heparin sodium was purchased from Beijing Yaobei Biological and Chemical Reagents Company (China).

### 2.3. Animals

Six male beagle dogs, weighing at 10-12 kg, from Tongli Laboratory Animals Center (Beijing, China) were adopted in the animal experiments. The Experiments were carried out in accordance with international accepted guidelines on laboratory animal use, the Guide for the care and use of laboratory animals (Institute of Laboratory Animal, Washington, D.C. National Academy Press 1996), and the protocols were approved by the Beijing Animal Care Committee (Beijing, China).

### 2.4. Preparation of blood samples

Solid phase extraction (SPE) cartridges were preconditioned by passing through 2 ml of methanol followed by 2 ml of water before loading. 0.5 ml plasma sample was applied to solid phase extraction cartridge (1 ml, packed with 100 mg of 40 μm octadecyl silica) and drawn through by gravity. The cartridge was washed chronologically with 2 ml of water, 2 ml of 20% (v/v) aqueous methanol solution, and 2 ml of methanol. The final methanol eluate was collected and 10 μL of internal standard was added to it. The eluate was thereafter evaporated at 60°C to dryness under a stream of nitrogen. The residue was dissolved in 100 μL of 50% (v/v) aqueous methanol solution. A 20 μL sample was injected into the HPLC system for analysis.

### 2.5. Preparation of normal and bio-adhesive tablet of PNS

We prepared normal tablet and bio-adhesive tablet according to our previous patent (Patent number: ZL200510073274.4). The ratio between PNS and other excipients in our two kinds of tablets was PNS: chitosan: other excipients = 60:40:100. Based on the standard of commercial XUESAITONG tablet, the dosage of PNS in our both bio-adhesive and normal tablet was 50 mg. We homogeneously mixed PNS with other excipients except chitosan after sifting, and used ethanol to wet-granulate particles. The particles passed through 20-mesh sieve were dried at 60°C. Then they were mixed with chitosan and magnesium stearate and were compressed into tablets by using a single-punch tablet press. Normal tablet was prepared by the similar ratio and method, without adding chitosan.

### 2.6. Administration of PNS and sample collection

According to our double cycle and crossover experimental design, normal and bio-adhesive tablets were administered orally at a dose of 90 mg/kg to six beagle dogs. All dogs were deprived of food but given free access to water for 12h before the experiments. Blood samples (3 ml each time) were collected via fore leg vein of dogs according to the specific time intervals of at 0, 0.5, 1, 2, 3, 4, 6, 8, 10, 12, 16, 24, 36, 48, 72h after administration. The plasma was separated after centrifugation for 10 mins at 3000 r·min^-1 ^and stored at -20°C for the further analysis.

### 2.7. In vivo pharmacokinetic study

All the data was processed by non-compartmental analysis using the Phoenix WinNonlin 6.1 (Pharsight, USA), and then was analyzed by SPSS software to judge its significance.

## 3. Results and Discussion

### 3.1. Validation of LC-MS/MS methods

**Figure 2:**
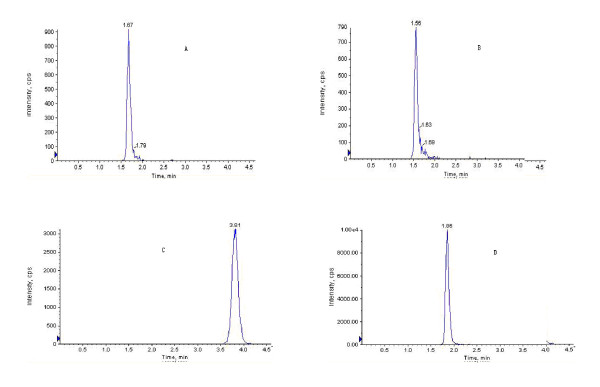
**Chromatograms under LC/MS/MS conditions for analyzing beagle dog plasma spiked with Notoginsenoside R1 (A), Ginsenoside Rg1 (B), Ginsenoside Rb1 (C) and internal standard glipizide (D)**. MS/MS transitions monitored in the positive ion mode were from m/z 1007 to m/z 423 for R1 (MW 932), from m/z 875 to m/z 423 for Rg1 (MW 800), from m/z 1183 to m/z 487 for Rb1 (MW 1108), and from m/z 494 to m/z 369.1 for internal standard. Intensity cps: ion intensity is in counts per second. The retention time is 1.67 min for R1, 1.56 min for Rg1, 3.81 min for Rb1 and 1.86 min for I.S.

**Figure 3:**
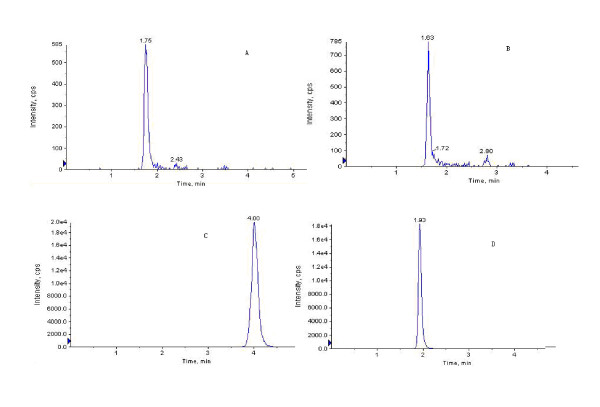
**Chromatograms under LC/MS/MS conditions for analyzing beagle dog plasma sample 2 h after oral administration of bio-adhesive tablet of PNS at dose of 90 mg/Kg. **Notoginsenoside R1 (A), Ginsenoside Rg1 (B), Ginsenoside Rb1 (C) and internal standard (D) were detected respectively. The retention time is 1.75 min for R1, 1.63 min for Rg1, 4.00 min for Rb1 and 1.93 min for I.S.

**Figure 4:**
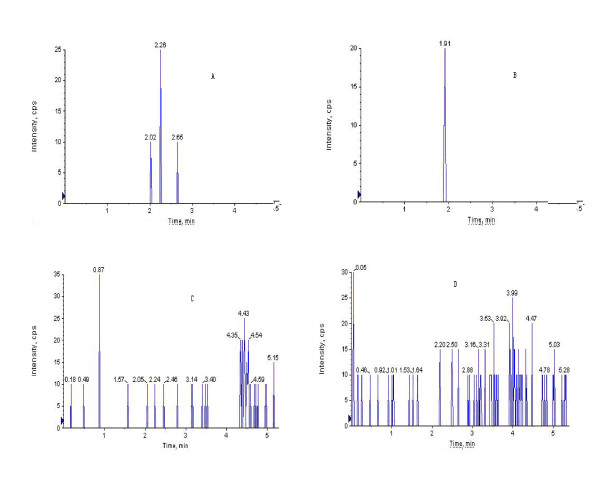
**Typical LC/MS/MS chromatograms of blank plasma of beagle dog under our detecting conditions**.

The specificity was evaluated by comparing chromatograms of blank plasma (from six different dogs), blank plasma spiked with R1, Rg1, Rb1, internal standard, and plasma samples after oral administration of by bio-adhesive tablet of PNS. As they were shown in Figure 2, 3, and 4, these chromatographic conditions revealed that there was no significant interfering endogenous peak observed within the period where R1, Rg1, Rb1 and internal standard were detected. Linearity of the calibration curves was found over the range of 2.8-140 ng for R1, 3.8-190 ng for Rg1 and 18-900 ng for Rb1. The equation of calibration curve for R1 was y = 0.0024x + 0.0068 (*r^2 ^*= 0.9967), for Rg1 was y = 0.001x + 0.0015 (*r^2 ^*= 0.9991); for Rb1 was y = 0.001x + 0.0197 (*r^2 ^*= 0.9982). The accuracy and precision were evaluated by quality control samples at low (2.8 ng/ml for R1, 3.8 ng/ml for Rg1 and 18 ng/ml for Rb1), medium (14 ng/ml for R1, 19 ng/ml for Rg1 and 180 ng/ml for Rb1), and high (140 ng/ml for R1, 190 ng/ml for Rg1 and 900 ng/ml for Rb1) concentrations which were prepared in the same way as calibration standards. The intra-batch accuracy and precision of the assay were assessed by quality control samples analysis at three concentrations in six replicates and the inter-batch was detected by analyzing them in three batches. The results met our requirement for detecting, and the detailed data have been published in our previous research article [15]. The relative recovery for R1 was 96.02% at 2.8 ng/ml, 104.02% at 14 ng/ml and 102.32% at 140 ng/ml; for Rg1 was 99.92% at 3.9 ng/ml, 102.40% at 19 ng/ml and 98.96% at 190 ng/ml; for Rb1 was102.67% at 18 ng/ml, 101.13% at 180 ng/ml and 105.00% at 900 ng/ml, the standard deviations were lower than 10.2%. The validated method was proved to be sensitive, reliable and the data demonstrated that this method was successfully applied to study the pharmacokinetics of PNS in beagle dogs after oral administration of PNS tablets.

### 3.2. Pharmacokinetic applications and plasma drug concentration-time curves of six beagle dogs

**Figure 5:**
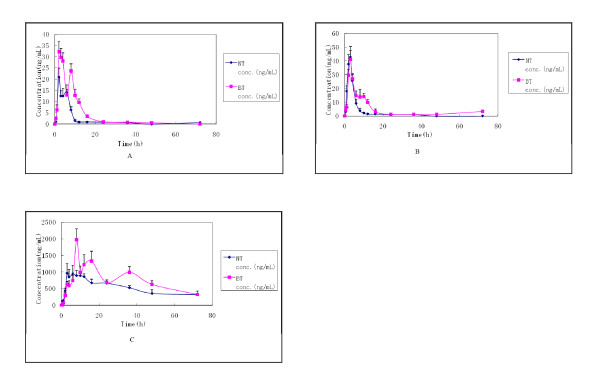
**Mean concentration-time profiles of Notoginsenoside R1 (A), Ginsenoside Rg1 (B) and Ginsenoside Rb1 (C) in beagle dog plasma after oral administration of two types of tablets, Normal tablet (NT) and Bio-adhesive tablet (BT)**. The dose of oral administration of PNS is 90 mg/kg (n = 6).

The highly developed digestive system of beagle dog is more similar to human and is more appropriate for pharmacokinetics study of medicine, especially for medicine passing through gastrointestinal tract [16]. In this study, we investigated the pharmacokinetics property of PNS in dogs, which is useful to prefigure its potential efficacy when it is administered to human being. The concentration-time curves of three components of six dogs were shown in Figure 5. All three concentration-time curves of bio-adhesive tablet of PNS shared a similar multiple-peak phenomenon. Our result was inconsistent with Xu's report [12], in which the rats' concentration-time curves of Rg1 and Rb1 were single-peak. In Xu's report, they failed to detect Rg1 in rats' serum after 24h, while we detected R1, Rg1 and Rb1 till 72h. The reason of this inconsistence might be the differences of administrating doses, analytical methods and formulation modifications between the previous and present studies. In our study, 90 mg/kg PNS tablet was orally administered to beagle dogs while the dose of PNS in documental reports was 600 mg/kg. Owing to our improved analytical method, the PNS blood concentration could be detected at nanogram level compared to the microgram level of Xu's report. By virtue of the improvement of our more sensitive detection method, we could reveal more veritable pharmacokinetics properties of PNS after oral administration.

Specifically, there were several potential factors contributed to multiple-peak phenomenon showed in all three ingredients' results: Because of the gastric emptying effect, drug got to small intestine where it was primarily absorbed nonsynchronously and hence entered the blood at different time; PNS was absorbed at different positions in gastrointestinal tract and each position had different absorption rate; Enterohepatic circulation: the two or more peaks occurred in three concentration-time curves might be caused by the hydrolytic effects of some bacterial enzymes or the influence that bile released intermittently during the process of re-absorption of PNS; Moreover, according to previous pharmacokinetics research, R1, Rg1 and Rb1 had a great distribution in several organs, especially in kidney, heart and liver [17-19]. Thus their redistribution effect might also be another possible cause for the multiple-peak phenomenon occurred in our study.

When we compared the pharmacokinetics property of Rb1 with R1 and Rg1, we found that there were more peaks in Rb1's concentration-time curve than that of R1 and Rg1. Besides, we detected high concentration of Rb1 in plasma till 72h, while the concentrations of the other two ingredients in plasma are quite low after 36h. It can be inferred that several possible reasons besides those we had discussed above may combine together to account for the different pharmacokinetics property of Rb1: In Han's research, the protein-binding rate in plasma for Rb1 (80.11~89.69%) was considerably higher than Rg1(6.56~12.74%), the protein-binding effect could prolong the existence of Rb1 in plasma, and the process that Rb1 dissociated from protein in plasma to release uncombined ingredient maybe related with more peaks in Rb1's concentration-time curve. Moreover, Rb1 might be absorbed in colon. Although previous study reported that Rb1 was degraded evidently in large intestine [20], chitosan was able to improve the stability of PNS, especially Rb1, due to its ability to improve the distribution of intestinal microorganisms, to inhibit the biological synthesis at the surface of bacteria's cell membrane and damage the transmission of energy crossed through it. Based on such mechanisms, chitosan reduced the degradation of Rb1 and enabled it to be absorbed in colon. Additionally, other ingredients in PNS might affect the absorption of Rb1. Considering of the experimental condition and animal-protectionism, we only used 6 beagle dogs in this study. So it was unreasonable to deny the possibility of individual difference between dogs as well as its influence on the result of our pharmacokinetics study. Using more beagle dogs might enable the result more persuasive, but it was not covered by this current study. The pharmacokinetics property of Rb1 was very complex and should be subjected to further study.

### 3.3. The relative bioavailability and other pharmacokinetics parameters

The parameters of AUC for R1, Rg1 and Rb1 between normal tablet and bio-adhesive tablet were increased remarkably (P < 0.05), and the relative bioavailability after oral administration of bio-adhesive tablets is 204.53% for R1, 152.73% for Rg1 and 150.50% for Rb1. This result (Table 1) indicates that bio-adhesive formulation considerably improved the oral bioavailability of PNS. The parameter of *T_max _*for R1, Rg1 and Rb1 was significantly increased (P < 0.05). The parameter of *C_max _*for R1 and Rb1 between normal tablet and bio-adhesive tablet was increased remarkably (P < 0.05), which reveals that there was a process of accelerating release or improving assimilation. Though there was no statistical difference of *C_max _*for Rg1 between two kinds of tablet, its MRT was significantly prolonged from 6.40h to 11.54h (P < 0.05), which indicated that the improvement of AUC of Rg1 was attributable to a prolonged assimilation and slow release. It can be inferred that the increase of MRT for Rg1 was probably the result of prolonging drug's residence time in gastrointestinal tract based on chitosan's specific properties of polymeric cationic, gelling and adhesivity. Chitosan's expansibility *in vivo *might retard the release of Rg1, and Rg1's high protein-binding rate in liver maybe another possible reason.

**Table 1:** Pharmacokinetic parameters of Notoginsenoside R1, Ginsenoside Rg1 and Rb1 after oral administration of PNS normal and bio-adhesive tablets (50 mg/kg) to dogs (n = 6)

	R1		Rg1		Rb1	
	
	NT	BT	NT	BT	NT	BT
Tmax(h)	2.67	5.00*	2.33	3.33*	9.33	10.67*

Cmax(ng/mL)	27.83	47.69*	55.58	42.03	1090.45	2448.90*

MRT(h)	9.93	9.19	6.40	11.54*	26.38	26.68

AUC(ng/mL)Sh	132.11	270.21*	199.08	304.06*	35579.73	53546.15*

NT: normal tablet; BT: Bio-adhesive tablet

* P < 0.05 versus oral administration of normal tablet.

The improvement of AUC for R1 and Rb1 were more likely a consequence of increasing permeation of drug through intercellular space by reducing the trans-membrane electric resistance of epidermal cell. The permeation might be further increased by the electrostatic interaction between positively charged chitosan and negative charged cell membrane. The different spatial structures of three components might also account for the different mechanisms upon which they relied to enhance AUC.

The formulation modification of bio-adhesive tablet using chitosan might be closely related with the changes of pharmacokinetics properties of PNS. Chitosan was applied in our study based on its unique advantages: 1. Low oral toxicity and good biocompatibility that guarantee sufficient safety for using. Chitosan was degraded into low molecular oligosaccharide by lysozgme or NAG, such product cannot accumulate in internal environment. The final degradation product glucosamine was able to be absorbed by the organism [21-23]; 2. Property to control or prolong drug's release: the viscous property and expansibility of chitosan enabled it to retard the release of PNS [24]; 3. Increasing permeation of drug through intercellular space: chitosan was able to open the cell junction and to influence the mucous membrane reversibly [25]; 4.Improving drug's stability in gastrointestinal tract: PNS was surrounded by chitosan and thus was protected against damage in acid environment of gastrointestinal tract [26]. According to some reports, Rg1 and Rb1 were unstable in gastric fluid [27-29], 90% Rg1 and 80% Rb1 were degraded after 2 hours incubation in simulated gastric fluid [30]. The amino group of chitosan could interact with acidic components and then neutralized excessive gastric acid, thereby reducing the damaging effect PNS encountered in stomach and enhancing its bioavailability.

## Conclusions

In this study we used chitosan as a bio-adhesive material to prepare a modified formulation in order to improve the bioavailability of PNS after oral administration, the experimental result has revealed that our bio-adhesive tablet has significantly enhanced the bioavailability of PNS in beagle dogs, which indicates that this kind of formulation is helpful to improve the therapeutic effect of PNS and conducive to expand its clinical application.

## Abbreviations

LC-MS/MS: liquid chromatography-tandem mass spectrometry; HPLC: high-performance liquid chromatography; SPE: solid-phase extraction; MRM: Multiple reaction monitoring; IS: internal standard; LOQ: limit of quantification; RSD: Relative standard deviation; BT: bio-adhesive tablet; NT: normal tablet.

## Competing interests

The authors declare that they have no competing interests.

## Authors' contributions

FHZ processed and interpreted the data, performed the statistical analysis and drafted and critically revised the manuscript; CW carried out the pharmacokinetics study; ZCY conceived of the study, and participated in its design and coordination. All authors read and approved the final manuscript.

## References

[B1] Yao XH Li XJ *Panax notoginseng *saponins injection in treatment of cerebral infarction with a multicenter study Chin J New Drugs Clin Rem 2001 20 257 260

[B2] Yao XH Li XJ Protective effects and its mechanism of panaxatriol saponins isolated from *Panax notoginseng *on cerebral ischemia China Journal of Chinese Materia Medica 2002 27 371 373 12774330

[B3] Zhao GR Xiang ZJ Ye TX Antioxidant activities of Salvia miltiorrhiza and Panax notoginseng Food Chemistry 2006 99 767 774 10.1016/j.foodchem.2005.09.002

[B4] Wang N Wan JB Li MY Wang YT Advances in studies on *Panax notoginseng *against atherosclerosis Chin Tradit Herb Drugs 2008 5 787 791

[B5] Wang R Li YN Wang GJ Neuroprotective Effects and Brain Transport of Ginsenoside Rg1 Chinese Journal of Natural Medicines 2009 7 315 320

[B6] Yang CY Wang J Zhao Y Anti-diabetic effects of *Panax notoginseng *saponins and its major anti-hyperglycemic components J Ethnopharmacol 2010 130 231 236 10.1016/j.jep.2010.04.039 20435129

[B7] Lau AJ Toh DF Chua TK Antiplatelet and anticoagulant effects of *Panax notoginseng*: Comparison of raw and steamed *Panax notoginseng *with *Panax ginseng *and *Panax quinquefolium* J Ethnopharmacol 2009 125 380 386 10.1016/j.jep.2009.07.038 19665534

[B8] Jung HW Seo UK Kim JH Flower extract of Panax notoginseng attenuates lipopolysaccharide-induced inflammatory response via blocking of NF-κB signaling pathway in murine macrophages Journal of Ethnopharmacology 2009 122 313 319 10.1016/j.jep.2008.12.024 19162159

[B9] Chen YM Li XW Zhu GH Analysis of 103 cases with adverse drug reactions induced by *Panax notoginseng *saponins injection China Journal of Chinese Materia Medica 2010 35 237 239 20394303

[B10] Zhu CY Chen W Preparation and in vitro Evaluation of Panax Notoginseng Saponines ( PNS) Bioadhesive Tablets with carbopol Chin Pharm J 2007 142 1877 1880

[B11] Dodane V Vilivalam VD Pharmaceutical applications of chitosan Pharm Sci Tec Today 1998 6 246 53

[B12] Xu QF Fang XL Chen DF Pharmacokinetics and bioavailability of ginsenoside Rb1 and Rg1 from *Panax notoginseng *in rats J Ethnopharmacol 2003 84 187 192 10.1016/S0378-8741(02)00317-3 12648814

[B13] Han M Fu S Fang XL Compar ison between the character istics of absorption and pharmacokinetic behavior of ginsenoside Rg1 and ginsenoside Rb1 of panax notoginseng saponins Acta Pharmaceutica Sinica 2007 42 8 849 853 17944233

[B14] Li XY Sun JG Wang GJ Simultaneous determination of panax notoginsenoside R1, ginsenoside Rg1, Rd, Re and Rb1 in rat plasma by HPLC/ESI/MS: platform for the pharmacokinetic evaluation of total panax notoginsenoside, a typical kind of multiple constituent traditional Chinese medicine Biomed Chromatogr 2007 21 735 746 10.1002/bmc.813 17385805

[B15] Chen W Dang YJ Zhu CY Simultaneous determination of three major bioactive saponins of *Panax notoginseng *using liquid chromatography-tandem mass spectrometry and a pharmacokinetic study Chin Med 2010 5 12 10.1186/1749-8546-5-12 20331853 PMC2848657

[B16] He C Laboratory Animal Science 2006 >China Agricultural University Press

[B17] Wang SX Miao WL Fang MF Effect of borneol on the tissue distribution of notoginseng R1, ginsenoside Rg1 and Re in rabbits J Fourth M il Med Univ 2009 30 2750 2752

[B18] Deng ZJ Guo JW Yang M Advance of Pharmacokinetics on Panax Notoginsenoside and its Active Monomers Pharmacy today 2009 1 24 27

[B19] He CX The Distribution of Ginsenoside Rb1 in Mice 2010

[B20] Han M Studies on Oral Absorption of Panax Notoginsenoside(PNS) and Preparation of W/O Microemulsion for Oral Administration 2006

[B21] Lu FQ Cao ZS Zhuang ZX Biodegradation and Biocompatibility of a Chitosan Film Journal of Biomedical Engineering 1998 15 183 185 12548911

[B22] Kean T Thanou M Biodegradation, biodistribution and toxicity of chitosan Advanced Drug Delivery Reviews 2010 62 3 11 10.1016/j.addr.2009.09.004 19800377

[B23] Paul B The safety of chitosan as a pharmaceutical excipient Regulatory Toxicology and Pharmacology 2010 56 290 299 10.1016/j.yrtph.2009.09.015 19788905

[B24] Jameela SR Kumary TV Lal AV Progesterone-loaded chitosan microspheres: a long acting biodegradable controlled delivery system J Controlled Release 1998 52 17 24 10.1016/S0168-3659(97)00187-9 9685932

[B25] Illum L Farraj NF Davis SS Chitosan as a novel nasal delivery system for peptide drugs Pharm Res 1994 11 1186 10.1023/A:1018901302450 7971722

[B26] Liu LS Liu SQ Ng SY Controlled release of interleukin-2 for tumour immunotherapy using alginate/chitosan porous microspheres J Controlled Release 1997 43 65 74 10.1016/S0168-3659(96)01471-X

[B27] Akao T Kida H Kanaoka M Intestinal bacterial hydrolysis is required for the appearance of compound K in rat plasma after oral administration of ginsenoside Rb1 from *Panax ginseng* Pharm Pharmacol 1998 50 1155 1160 10.1111/j.2042-7158.1998.tb03327.x 9821663

[B28] Hideo H Jong HS Satoshi M Main ginseng saponin metabolites formed by intestinal bacteria Planta Med 1996 62 453 457 10.1055/s-2006-957938 8923812

[B29] Wang Y Liu TH Wang W Studies on the metabolism of ginsenoside Rg1 by intestinal bacteria and its absorbed metabolites in rat and human serum Acta Pharm Sin 2000 35 284 288

[B30] Han M Fu S Fang XL Screening of panax notoginsenoside water in oil microemulsion formulations and their evaluation in vitro and in vivo Acta Pharm Sin 2007 42 780 786 17882965

